# Dairy farmer, engagement and understanding of One Health and antimicrobial resistance - a pilot survey from the lower north island of Aotearoa New Zealand

**DOI:** 10.1186/s42522-024-00107-7

**Published:** 2024-08-01

**Authors:** Kurt Arden, Sarah M. Rosanowski, Richard A Laven, Kristina R. Mueller

**Affiliations:** 1 Veterinary Epidemiology, Economics and Public Health, Royal Veterinary College, Hawkshead Ln, Brookmans Park, Hatfield, UK; 2 Data Science, Digital Agriculture, Grasslands Research Centre, AgResearch Limited, Manawatu-Wanganui, Palmerston North, New Zealand; 3 School of Veterinary Sciences, Massey University, Manawatu-Wanganui, Palmerston North, New Zealand

**Keywords:** Aotearoa-New Zealand, One Health, Dairy Farmers, Antimicrobial Resistance, Antimicrobial Usage

## Abstract

**Background:**

Reducing antimicrobial resistance (AMR) requires a multidisciplinary One Health approach, which necessitates buy-in from all stakeholders. In Aotearoa New Zealand, where the dairy industry is one of the largest users of antimicrobials, there are ongoing efforts to optimise antimicrobial usage (AMU) to minimise the development of AMR. These include regulations around the veterinary authorisation of the use of antibiotics by farmers without the need for a specific prescription (“the RVM process”) and programmes such as the New Zealand Veterinary Association’s antibiotic ‘Traffic Light System’. The goal of this pilot survey was to develop and trial a questionnaire to determine how much Aotearoa dairy farmers understand about One Health, AMR, the RVM process and how their actions regarding AMU affect the wider environment.

**Methods:**

A 55-question semi-structured questionnaire was piloted on 15 dairy farms in the Lower North Island of Aotearoa New Zealand via an in-person semi-structured interview between September and November 2021.

**Results:**

None of the interviewed farmers could define the term One Health. However, the majority found the RVM process to be of use on their farm, although admitted they generally felt frustration regarding AMR, seeing it as a blockage to productivity, and lacked awareness regarding how their actions were related to its development. Of the farmers interviewed over half had not heard of the traffic light system, and of those who had, one admitted they refused to adhere to it.

**Conclusions:**

This survey’s novel findings have highlighted that there are notable gaps within dairy farmer understanding of AMU, AMR and One Health as well as highlighting that veterinarians could do more to keep their clients informed of their important role within One Health. There is still a lot more work to do with regards to vets, farmers and industry representatives working together to embrace One Health. Simple solutions would be to encourage farmers returning unused drugs to their veterinarians for correct disposal and to actively engage farmers further regarding AMU and AMR, so that these end-product users do not feel disconnected from the process.

**Supplementary Information:**

The online version contains supplementary material available at 10.1186/s42522-024-00107-7.

## Introduction

### Antimicrobial resistance

The development of antibiotics is one of the most important advances in modern medicine [26]. However, increasing antimicrobial resistance (AMR) threatens this progress [34]. AMR is a complex multifaceted global issue, which threatens both animal, human and ecosystem health, as well as food equity and global food security [9, 12, 16]. AMR can be defined as the result of microbes, such as bacteria, developing mechanisms that prevent antimicrobial agents such as antibiotics from killing them [5] to an antimicrobial agent to which they were originally sensitive [28]. This is a natural process which ensures the long-term survival of microbial species to their environment. However, in recent years antimicrobial use (AMU) in both humans and animals has increased. This overuse of antimicrobial compounds has led to AMR becoming a public health and ecological hazard [32] and is now categorized as one of the top ten global public health challenges by the World Health Organisation (WHO) [66]. In 2019, five million human deaths were linked to AMR, with 1.3 million of those directly caused by antimicrobial resistant bacteria [48]. Furthermore, AMU is not just causing AMR issues in human health, AMR is also well documented in wild, companion and production animals [23], as well as directly impacting plant health [41].

### AMR as a One Health problem

Due to the interconnectedness of the human, animal and environmental impacts of AMR, it is considered to be a ‘One-Health’ problem, as tackling the complexity of the drivers impacts of AMR on microbiological populations, is best achieved by a multi-sectional co-ordinated response [9]. One Health is defined as an “*integrated, unifying approach that aims to sustainably balance and optimize the health of people, animals, and ecosystems*”, which “*recognizes the health of humans, domestic and wild animals, plants, and the wider environment (including ecosystems) are closely linked and interdependent*” [1]. This definition incorporates the key concepts of human, animal and ecosystem health, as well as addressing the anthropogenic factors which exert an effect on the wider environment [67]. In 2022, the quadripartite comprising of the Food and Agriculture Organization of the United Nations (FAO), the United Nations Environment Programme (UNEP), the World Organisation for Animal Health (WOAH, founded as OIE), and the World Health Organization (WHO) created the One Health High-Level Expert Panel (OHHLEP) to provide technical and scientific advice on One Health issues. Antimicrobial resistance is one such issue of importance to the OHHLEP and they worked alongside other One Health focused bodies such as the Quadripartite Global Leaders Group on Antimicrobial Resistance (GLG-AMR), to create the One Health joint action plan with one such goal to be curbing the silent pandemic of antimicrobial resistance [38].

### One Health and AMR in Aotearoa New Zealand

Aotearoa New Zealand (Aotearoa) has a relatively low rate of AMR in people, compared to other high income countries [62]. However, the human data also shows a steady rise in resistance genes in Aotearoa, with a range of factors including inappropriate antimicrobial use, increased transmission of resistant organisms, importation of multidrug-resistant bacteria, and increased viability of resistant organisms thought to be responsible for this increase [68]. With regards to animal health data, the prevalence of AMR in livestock is believed to be currently low in Aotearoa; one key reason for this is that use of antibiotics in livestock is also comparatively low to other nations [6]. Aotearoa is one of the three countries in the Organisation for Economic Co-operation and Development with the lowest use of antibiotics to treat livestock disease [25]. However, the true burden of disease due to AMR within Aotearoa’s biosphere is not currently clear and the role of the natural environment in AMR transmission is especially unclear [52].

Biosecurity and AMR are intrinsically linked [61] and in Aotearoa one of the best forms of biosecurity is the geographic isolation of the country [59]. However, on farm biosecurity practices remain variable [18] and recent exotic diseases outbreaks highlighted that border control is not infallible [30]. Improved farmer understanding of how a One Health approach can assist them is essential and will become even more crucial as climate change driven by anthropogenic factors is intimately linked to AMR [33], for example cyclones and other severe weather changes are becoming more frequent in Aotearoa [21]. As such, targeted biosecurity mitigation strategies are needed on farms to prevent the increasing risk of AMR.

The global definition of One Health was formally accepted by the international community in 2022 [38]. However, the multidisciplinary movement first started appearing around the turn of the twenty-first century [4]. Recent global attention was brought on by the coronavirus (COVID-19) pandemic, with governments across the globe including The Group of Seven (G7) calling for a One Health expansion within regulatory bodies [17]. This global attention was one of the driving forces behind the creation of this survey, to determine whether One Health as a term and as method of action was reaching the key stakeholders responsible for its success.

### Regulatory control and assessments of AMU in Aotearoa

As documented by Pattis et al. [52], the use of antimicrobials in animal systems is low in Aotearoa. One reason for this low use is strong regulatory control of AMU. In Aotearoa, the majority of antimicrobial agents can only be used to treat livestock disease when prescribed by a veterinarian, i.e., they are restricted veterinary medicines (RVM). The RVM process is regulated under the Agricultural Compounds and Veterinary Medicines Act 1997 (ACVM) and associated regulations such as the 2015 requirements for authorising veterinarians [44]. Under the ACVM, all antibiotics are classified as RVMs, however livestock farmers can have autonomy over on-farm drug administration and use and store RVMs on farm without primary veterinary supervision. This is because veterinarians can authorise non-veterinarians whose animals are under their care to hold RVMs for future use through a consultation process. Individual veterinary examination is not possible on all livestock cases in Aotearoa due to a multitude of sociological and geographical reasons. As such the authorising veterinarians are able to authorise non-veterinarians so long as they are satisfied that the non-veterinarian has enough current knowledge of the health of the animals to support the authorisation, i.e., they must ensure that the ongoing use and choice of RVMs remains appropriate and necessary, and that any person who will administer the RVM can competently carry out the authorising veterinarian’s instructions. Following this consultation, RVMs can be authorised to be used in line with specific instructions on specified animals in specific situations. The aim of the process is to manage risks associated with the use of RVMs, such as risks to animal welfare, public health and trade, without overburdening farm staff further by requiring veterinary examination of every animal before treatment. This is especially apparent for herd/flock level treatment plans such as dry cow therapy (DCT) [45].

In addition to these statutory requirements, other resources are also available to veterinarians to aid with prudent antibiotic use. One crucial resource, whilst not statutory but is considered nationally best practice, is the antibiotic ‘Traffic Light System’ developed by the New Zealand Veterinary Association (NZVA) [36] (Table 1). This is a modified, nationally relevant, less restrictive version of the WHO highest priority critically important antibiotic (HPCIA) classification system [65], which also incorporates aspects of the WOAH’s list of antimicrobial agents [69]. The role of the traffic light system is to provide practical guidelines for veterinarians regarding which drugs they should use as first line antimicrobials, and for farmers to be aware of what they can expect to receive with regards to their animal’s treatment.

**Table 1:** The NZVA Traffic Light System of antibiotics as provided to dairy farmers in Aotearoa [50]

New Zealand Veterinary Association Antibiotic Classification ‘Traffic Light’ System		
Green antimicrobials	Orange antimicrobials	Red antimicrobials
• Procaine penicillin • Penethamate hydriodide • Tetracyclines	• Aminoglycosides • Semi-synthetic penicillin’s (ampicillin, clavulanic acid, cloxacillin) • 1st and 2nd generation cephalosporins • Lincosamides • Potentiates sulphonamides	• 3rd and 4th generation cephalosporins • Fluroquinolones • Macrolides
Green is recommended against known susceptible organisms and should not be used in situations where efficacy is in doubt. Relevant to human therapy	Orange have specialised features and are of more critical relevance to human therapy	Red are reserved for treatment of refractory conditions, where diagnosis and evidence indicates their essential need, or where efficacy of other classes is limited. Highest Priority Critically Important antibiotics for human therapy

The NZVA traffic light system is the official technical advice provided to veterinarians regarding HPCIA use in Aotearoa from the Veterinary Council of New Zealand (VCNZ). If implemented effectively, both the RVM process and the Traffic Light System should reduce the development of AMR in livestock in Aotearoa. However, to be effective in reducing AMR, they require buy-in from the end user, i.e., farmers need to understand the value of the RVM process and the Traffic Light System as well as understanding the importance of a One Health approach. This is perhaps most important on dairy farms which are the largest (in total volume) users of antibiotics in Aotearoa, with DCT being the largest, per volume, use of antimicrobials [25].

Regulatory oversight of AMU and AMS is an essential tool to combat AMR. Some countries, such as Australia, have a technical advisory group that maintain a current list of importance ratings and summary list of antibacterial uses in human and animal health [3]. It is of crucial importance to translate international approaches into nationally relevant guidelines, a true One Health approach utilises human and animal health as well as environmental surveillance to create locally pertinent specific objectives to an individual country’s animals and production systems [20].

The third measure of AMS to be explored in the survey is the independent party annual shed inspection assessment, which is the milk purchasers annual supply audit. Every milk-supplying dairy farm is required to have an inspection each year by an independent party, known as a shed inspection, to ensure that the farm is meeting their milk purchasers requirements for supply [56]. Currently the assessment covers food safety and quality, animal health and welfare as well as aspects of environmental health. Antimicrobial storage forms part of the animal health and welfare assessment [13], which is separate to the veterinary RVM process, however there is overlap [43]. This is an important AMR and AMU audit as it assesses farming practices associated with antimicrobial storage and effluent management which is becoming increasing relevant to better understand the role of the environment and water contamination (with antibiotics and resistant pathogens and elements) in this One Health issue [52].

The combination of RVMs and shed inspections highlight Aotearoa’s forward thinking approach to AMS and demonstrates the regulatory oversight of key stakeholders, veterinarians and dairy farmers, within this important area [47]. The New Zealand Antimicrobial Resistance Action Plan is the current guiding official document for best practice regarding AMS in New Zealand covering all health care professionals [44].

### AMR and its implications for Aotearoa

Historically, dairy farming has involved surplus use of antibiotics [55], which has led to an increase in AMR [27], with the large quantities of antibiotics utilized in DCT being a contributing factor [19]. The dairy industry is worth over $20 billion dollars to the Aotearoa economy and along with other members of the food and fibre sector makes up 11% of the nation’s gross domestic product (GDP) [46]. AMR possess a serious threat to Aotearoa dairy sector, not only as a source of economic loss due to decreased treatment efficacy but also as a source of human and environmental antibiotic resistance mechanism distribution [19]. However, antimicrobial usage (AMU) in dairy cows in Aotearoa is low, compared to international standards [6]. There is some evidence which suggests a trend of decreasing prevalence in some pathogenic organisms exhibiting AMR, for example McDougall et al. [37] reported a reduction in Staphylococcus aureus isolates resistant to penicillin. Despite this, there has been noted variation in farmer habits regarding AMU both spatially and temporally [6], and these factors are worthy of consideration, as Burgess and French [7] discuss that the risks of AMR in dairy cattle can lead to reduced market access of Aotearoa products of animal origin. The RVM, as a form of regulatory oversight, provides assurance to international trading partners of the safety of Aotearoa’s primary products.

Aotearoa’s reliance on its primary industries means that the downward trend in antimicrobial usage and resistance from on-farm sources must continue, otherwise Aotearoa’s ‘‘clean and green’’ [39] image may be easily tarnished upon the international stage by inappropriate corporate activity [31]. This will require producers to engage with the One Health model and to work with their veterinarians, through the RVM process and by following the NZVA traffic light system, to optimise antibiotic use. In Aotearoa, farmers do not receive formal education with both AMR or One Health, unless they have undertaken higher education study, some resources do exist, however engagement is voluntary [49].

### Survey aim

The aim of this pilot survey was to examine dairy farmers’ opinions, understanding and awareness of three on farm current AMR and AMU interventions and how the OH approach is important to reduce AMR and transmission of resistant microbes. The three on farm interventions investigated in the survey included: veterinary RVM process, NZVA traffic light system and annual shed inspection assessments.

## Materials and Methods

### Participants

A cross-sectional pilot study involving fifteen Dairy farmers from the Lower North Island of Aotearoa was conducted between September and November 2021. All individuals who owned or managed dairy farms, whilst being registered and having an active RVM from the conducting veterinary practice, were invited to participate in the survey, and all agreed.

### Data collection and handling

A semi-structured questionnaire, focused on information deemed essential by One Health experts at Massey University, such as AMR, AMU, AMS, and medicine storage and administration, in addition to One Health approaches of the veterinary practice the farmers were registered with, was designed (see appendix). Additional information was collected, as the questionnaire also incorporated aspects of customer centricity allowing the farmer to provide feedback to the veterinary practice, such information irrelevant to this study was used separately. The questionnaire was tested with two of the fifteen dairy farmers to assess farmer interpretation of the questions, the time to complete the questionnaire and ease of transcription. The questionnaire was not refined following as no changes were deemed necessary.

The questionnaire was delivered directly to the dairy farmers, on-farm during a routine veterinary visit. All interviews were conducted by the first author and the interviewer was one of the regular veterinarians for the farm. However, the interviewer was not providing the annual RVM appointment in the year of data collection, this was deliberately chosen to minimise data collection issues. Interviews were transcribed by the interviewer at the time of interview if clarity was required the question was repeated to the farmer and they were asked to confirm if this was their intended answer**.** The interviewer was trained in conducting interviews as part of prior research projects. A semi- structured approach was chosen to enable a greater success of completion compared to online questionnaires. To further farmer engagement the questionnaire was undertaken as part of routine visits to the dairy farm and took up to one hour to complete, depending on farmer enthusiasm with answers.

### Data analysis

Closed question responses were described as counts and percentages. For each question or question set, responses could be categorised as “I don’t know” or the farmer indicating some knowledge as a binary (yes or no) and the number of farmers in each category reported. Data from open-ended questions were treated as qualitative. Where appropriate, responses from related qualitative questions were grouped in the questionnaire subsections e.g. Antimicrobial resistance or RVM process etc., and analysed together, following a content analysis approach [22].

Content analysis is a technique for reducing the content of verbal data into more defined categories to enable interpretation. For the current study, the content analysis approach was used for questions where farmers provided some elaboration within their answer. The content of each answer was distilled based on the interpretation of keywords identified within each farmer’s response and similarities between keywords and farmers were noted. To ensure anonymity, farmers were identified by a number and direct quotes are provided in italics, where appropriate.

All data were stored and handled in Excel [40] and Stata IC version 18 [58]

## Results

### Description of the participants

Demographic data of the survey participants is presented in Table 2. All participants were the person stated on the RVM and had legal responsibility for drug usage, storage, and disposal on the farm of operation. The range of head of cattle on the farms used in this study was from 160 – 700, all cattle were extensively raised, and grass fed with silage and concentrate provided as necessary.

**Table 2:** Demographic data of the survey participants

Demographic Variable	Number of participants
Farm ownership model	
Sharemilkers (50/50-part ownership)	4
Farm managers (employees)	3
Farm owner (owner of farm and land)	8
Age of participant	
30 to 40 years old	3
40 to 50 years old	4
50 to 60 years old	3
60 years plus	5
Ethnicity of participant	
Pakeha (White New Zealander)	13
Māori—Ngāti Kahungunu	1
European	1
Highest level of qualification	
High School graduates	6
Undergraduate qualification	8
Post-graduate qualification	1

### One Health and AMR

Nine of the 15 farmers stated that they did not know anything about One Health, whilst six attempted to define it. The majority of farmers that did discuss about their understanding of One Health commented from the perspective or importance of animals within the triad. The breadth of this topic was captured by several respondents by stating "everything" or "incorporating the whole herd". Thirteen farmers stated that they knew what AMR meant, with twelve providing further elaboration. Nine farmers reused the term "resistance" within the discourse about their understanding. All farmers who elaborated referred to either drugs, antibiotics, or specific antibiotics, or only mentioned bacteria. One farmer stated it was the animal building immunity to drugs.

### Awareness of NZVA Traffic Light System

Four farmers had not heard of the NZVA “Traffic Light System”, whilst one of those who had, reported that it was not relevant to them or their farming practice. Three farmers mentioned the connection between Red (restricted use) antimicrobials and human health. Of the ten farmers who reported some understanding of the Traffic Light System, five stated that they would consider the system when they perceive a need for antimicrobial treatment on their farm. One farmer would rely on their veterinarian’s advice and one farmer noted that they would consider it now, following the interview. Five farmers stated they never considered antimicrobial resistance when treating stock and no farmers considered One Health when opting for antimicrobial treatment.

### RVM and RVM process

Farmer 14 summarised the sentiment and understanding of the farmers with regards to the RVM – *"What drugs I can and can't use on farm."* [ID 14].

In general, farmers noted the importance of the RVM process for commercial compliance, and discussed more specifically about which antibiotics could be used on farm without a veterinarian present. Two farmers responded that the purpose of the RVM was to help them keep track of what medicines they were using, to ensure proper use and/or prevent overuse. Uniformly, farmers noted the importance of the veterinarian in this process, through monitoring, although this could be due to bias as it was veterinarians conducting the questionnaire. Most respondents’ answers focused on an individual or farm-level, with only one farmer noting that the RVM process was a legal obligation. Most farmers said they understood the difference between the RVM and the RVM process, with the consultation being the discussion around RVMs to document the veterinary medications that would be required going forward. Two farmers noted that they did not believe that the RVM process added value to their farms. One of these was an organic farmer, who stated that they did not use many veterinary medicines, whilst the second farmer stated that it was a “*pointless exercise*”. Of the farmers that found the RVM did add value, farmers noted “*improving outcomes*” [ID 15], “*keeping up to date*” [ID 12; ID 1] and as an opportunity to seek “*veterinary advice*” [ID 14].

Regarding questions on whether the shed inspection should or could form a part of the RVM consultation, farmers were unanimously opposed to this suggestion and stated that veterinarians should not work more closely with shed inspectors. The reasons for this included the expertise and knowledge of veterinarians with regards to veterinary medicines and herd health, as opposed to a lack of perceived expertise from shed inspectors; “*don’t have great knowledge of drugs, best left to vets*” [ID 2]. Other farmers appreciated or valued the separation between the RVM consults and shed inspections for confidentiality purposes especially regarding storage and record use.

Including the organic farm, eleven farmers had no veterinary medicines on their farm that were not on their approved RVM list. Three farmers had medicines that were not on their list of authorised RVMs present on farm during the interview. In all three cases this was Vibrostrep (streptomycin/dihydrostreptomycin Virbac, Hamilton, New Zealand), an orange light antibiotic,. In addition, one farmer had Excede LA (ceftiofur, Zoetis, Auckland, New Zealand) on their farm. These antibiotics were not on the authorised RVM list for any of the 15 farms but were excess unused product from a separate prescription (after veterinary consultation) to treat one or more individual animals. All three farmers had kept the excess unused product on farm rather than returning it to the veterinary practice. Thirteen farmers reported having veterinary medicines not on the RVM list on farm in the past year, but these were present on farm at the time of the interview. The reported non-RVM drugs were long acting amoxycillin (Betamox LA, Norbrook, Auckland, New Zealand; four farms), Vibrostrep (eight farms), oral sulphonamide suspension (Scourban Plus, Elanco, Auckland, New Zealand; one farm), 10% marbofloxacin (Marbocyl, Vetoquinol, Auckland, New Zealand; one farm), 16% marbofloxacin (Forcyl, Vetoquinol, Auckland, New Zealand; two farms) and cefitofur crystalline free acid (Excede, Zoetis, Auckland, New Zealand; one farm). These antibiotics had all been authorised for use on-farm for specific case treatments outside of the yearly RVM consultation.

The veterinary medicines currently on farm at time of interview are described in Table 3. All non-organic farms had at least one antimicrobial on their approved list, several farms had non-RVM drugs on farm under separate active prescription for specific cases.

**Table 3:** Veterinary medicines reported by farmers to be on their farm, trade name, active ingredient and NZVA traffic light categorisation

Trade name	Active ingredient	Type of Drug	Traffic light category	On RVM	Number of farms
Bivatop	Oxytetracycline	Antibiotic	Green	Yes	6
Orbenin	Cloxacillin	Antibiotic	Green	Yes	8
Orbenin eye	Cloxacillin	Antibiotic	Green	Yes	2
Intracillin 300	procaine penicillin and benzathine penicillin	Antibiotic	Green	Yes	13
Intracillin (LA)	procaine penicillin	Antibiotic	Green	Yes	3
Pharmacillin 300	Procaine Benzylpenicillin	Antibiotic	Green	Yes	2
Penetheject	Penethacillin	Antibiotic	Green	Yes	5
Depocillin	procaine penicillin	Antibiotic	Green	Yes	1
Clavulox L.C	200mg Amoxicillin 50mg Clavulanic Acid Amoxicillin, Clavulanic Acid and Prednisolone	Antibiotic	Orange	No (on separate prescription)	3
Kelacef	Ceftiofur	Antibiotic	Red	No (on separate prescription)	1
Excede LA	Ceftiofur	Antibiotic	Red	No	1
Vibrostrep	Streptomycin/dihydrostreptomycin	Antibiotic	Orange	No	

### The use of veterinary medicines on farm

Excluding the organic farm, one farmer reported that they had not used veterinary prescription medications in the previous year. Four farmers stated that they used medications one to two times per year, three farmers used them once every six months, four farmers once every three months, one farmer used them less than ten times per year, and one farmer noted using them rarely.

Of the 15 farmers, two refused to answer the question regarding how they dispose of veterinary drugs on their farm. Seven farmers disposed of expired veterinary medications in the bin or by burying them. Four farmers reported giving the drugs back to the veterinarian to dispose of one farmer gave them to the dairy inspector and one farmer stated they kept them on farm to use the remaining drugs on other animals as they deemed necessary and then binned the containers. Twelve farmers recorded their veterinary medicine usage within an hour of use, two on the same day, and one within the week of usage. A variety of measures to record usage was implemented including a whiteboard (7 farms), using a farm diary (5 farms), and/or using an app (11). Eight farms used both a paper and an electronic record management system.

All farmers reported knowing the application method of the veterinary medicines that they were using. The respondents noted that if they did not know the application method, then they could read the bottle, product label or data sheet (4), review the RVM (4), or use “Google” (3) or ask their veterinarian (2). One farmer did not know how they would find out the route of administration if they did not already know. Farmers reported that the reason that the application methods were different for different veterinary medicines was due to efficacy (6), different routes being for targeted treatment, e.g. “*broad spectrum, specific areas*” [ID 8] (7), one spoke of time frames for effect or absorption of the medicine, and one farmer did not know. All farmers indicated that dose rates were necessary to calculate based on the weight or size of the animal (5), that the correct dosage was important (8) and that under or overdosing may be ineffective (5).

With regards to off label use, nine farmers identified “all of the above” as the definition for off-label use with the remaining farmers unsure of how it should be defined. Farmers defined withdrawal times variably covering aspects of food product contamination and affecting the manufacturing process. Only two farmers raised the risks associated with antibiotics from animals being consumed by humans and the development of AMR. Half of the farmers stated that they remembered the withdrawal times, whilst the rest would either review the bottle, the label, the datasheet or the RVM paperwork if they were unsure.

### Antimicrobial usage

As part of the questionnaire, farmers were asked about their AMU habits and what they routinely used on their farm and how frequently. Six farmers, including the organic farmer, did not use antimicrobials as their first treatment option. Seven farmers considered antimicrobials as the first treatment on a case-by-case basis and one farmer reported using ceftiofur as the first treatment for a sick animal. One farmer reported requesting culture and sensitivity testing on every occasion from blood and milk samples. Two farmers requested testing on a case-by-case basis for milk and one routinely used the farm-side Mastatest (Mastaplex Ltd, Dunedin, New Zealand). The remaining farmers requested culture and sensitivity testing infrequently for milk samples.

## Discussion

This pilot survey explored the practices and understanding of a small subset of dairy farmers based within the Lower North Island of Aotearoa. This study is, as far as the authors are aware, the first time globally dairy farmers have been asked to define their understanding of One Health, what it means to them and their role within it. The survey also asked farmers to critically evaluate the RVM process and explain the role of NZVA’s traffic light system, demonstrating that a lot more work needs to be undertaken in this area by both regulatory bodies and practicing veterinarians, to communicate the importance of a One Health approach and AMR to dairy farmers. The World Health organization has categorized AMR as one of the top threats facing public health, creating the global action plan on AMR and the HPCIAs watch list [65]. However, significant AMU reduction, a key cornerstone of the plan, is unlikely to be achieved within the dairy industry without successful cooperation from farmers [36].

### One Health and AMU in Aotearoa

In this study, One Health was not found to be fully understood or acknowledged by the farmers interviewed. However, just over half of the farmers interviewed, eight respondents, made connections between AMU and AMR, with five showing limited knowledge or concern of AMR. The responses to this question focused primarily on the human health aspect of AMR which is interesting as it shows a level of engagement within the One Health triad and that farmers are engaging with One Health topics even if they are not aware of it, and suggests that the work of regulatory authorities, veterinarians, shed inspectors etc. is at a minimum making farmers aware of the importance of their role in public health.

The results of this study are comparable to the responses reported by McDougall et al. [36] who evaluated farmer attitudes to AMU across both the North and South Island of Aotearoa. Our results support the conclusion that there is not a great variation in behavioural patterns of dairy farmers around the topic of AMU in Aotearoa. This is consistent with findings from England and Wales Jones et al. [29], which reported that the intention to reduce future antibiotic use was only very weakly correlated with current and past antibiotic use practice. Interestingly however, Jones et al. [29] reported that desire to reduce costs, where this would not impair the health and welfare of their stock, was found to be a strong driver of farmer behaviour in regard to AMU, yet in the present study cost was not mentioned by any of the farmers interviewed. Some of the farmers in the current study openly displayed almost habitual AMU practice such as routinely using ceftiofur for conditions outside of its labelled use. Another area of difference from Jones et al. [29] was that English and Welsh farmers were concerned with wider societal concerns about inappropriate antibiotic use, however this was not noted in this study with multiple interviewees usually stating a preference for a drug they believed would “*fix*” the animal.

The lack of farmers understanding of One Health, although not surprising, is concerning as this has progressed from more than just a buzzword [57] and with its larger definition encompassing the multitude of facets essential to prolonged survival of life on earth [67] highlights the need for radical change within community mindsets. Within One Health, AMU and AMR are integrally linked to long term sustainable utilisation of livestock and the recorded responses demonstrate a knowledge gap in this key understanding. Furthermore, the farmers interviewed in this study lacked full comprehension of AMR, although they self-stated they knew what the term meant, when asked for further elaboration, they reused the term “*resistance*” within the discourse about their understanding. This repeating of the question in the answer does not necessarily demonstrate true understanding of the concept. This is further reinforced as when interviewees were asked to expand on this answer none were fully able to, one suggested that the “*animal built immunity to the* drugs” and one farmer noted that they always finish courses of antimicrobials as this is what they do when they themselves take antibiotics but could not explain why.

### Responses to Regulatory control in Aotearoa

Interestingly, farmers unanimously did not want greater cohesion between veterinary practices, the RVM process and shed inspections. The reasons for this varied greatly, yet notable comments suggested that veterinarians held the expertise with regards to veterinary medicines and that shed inspections were simply a review carried out to satisfy regulations. For some of the respondents this view of shed inspections was consistent with the view that the whole of the RVM process was simply about regulation. However, shed inspections form an essential component of protecting a country’s international reputation [14] and are therefore integral. These opinions which undermine both shed inspections and the RVM process further reinforce the lack of farmer understanding of One Health, what it is and why it is important. This perhaps reflects the lack of a multifaceted One Health problem approach [64] and only by utilising a multifaceted approach across disciplines will this global issue be challenged. To achieve this goal a true partnership with both the farmers and the shed-inspectors should be fostered to avoid farmers feeling like they are being overly “monitored” and “sanctioned”.

There was a wide variety of answers provided by farmers for how veterinary medicines, including antibiotics, were disposed of. Several answers indicated illegal methods of disposal, further illustrating the need for a targeted One Health approach to prevent antibiotic leaching into the environment and the ensuing effects. Several farmers openly admitted keeping left over antibiotics from finished treatments to use on other animals when they were sick with no veterinary consultation, a behaviour that has been linked to promotion of AMR [51]. No farmers made the connection between drug residues and disposal and One Health, and very few made connections with regards to AMR, which is contradictory to their linkage to public health when defining what AMR is. Simple solutions exist such as the farmer can save the unused antibiotic and return it to the veterinarian at the next visitation for the vet to dispose of, involving no cost to the farmer, however this was not done by any of the farmers surveyed. These results are concerning as farmers are the end-users of the drugs and their choices have wide-reaching implications, veterinarians have a key role as gate keepers of these compounds, and it is their responsibility to ensure farmers are informed of the importance of their decisions. The results of this study regarding dairy farmer understanding of One Health show that more work is required before farmers on the ground understand how their actions affect society.

A quarter of the interviewed farmers had not heard of the Traffic Light System and didn’t see the value of the RVM as it was not relevant to them or their farming practice. There was no consensus among farmers who provided a response to the purpose of the Traffic Light System, with only three farmers making a potential connection between Red (restricted use) antimicrobials and human health. Veterinarians have a key role to play within the One Health approach and by utilising the NZVA traffic light system and using the RVM process as tools to combat AMR, the veterinary industry should be in a strong position to keep AMR at the low levels we currently see in large animal veterinary practice [25]. Worryingly, only five farmers stated that they considered antimicrobial resistance when treating stock. Unsurprisingly, as no farmer was aware of One Health, no farmers considered One Health when opting for antimicrobial treatment.

Interestingly there was a discrepancy between what antibiotics farmers reported having on farm, which were non-RVM drugs and what they physically had on farm. Thirteen farmers reported having veterinary medicines not on the RVM on farm, these were Betamox (Amoxicillin – orange light), Vibrostrep (streptomycin/dihydrostreptomycin – orange light), scourban (Sulphonamide – orange light), forcyl (Marbofloxacin – red light), Exceede (ceftiofur – red light) and Marbocyl (Marbofloxacin – red light). Farmers are able to have orange and red-light antibiotics on their farm with appropriate veterinary prescription, separate to their RVM. However, the non-RVM drugs listed by the farmers as being on their farm, is concerning, especially when compared with farmers stating they keep any left-over non-RVM drugs on their farm for future use. This is because these drugs were known to the farmers and include several red-light antibiotic (HPCIAs) such as Marbofloxacin and ceftiofur, Which Indicates farmer familiarity with red light antibiotics which should only be used under direct veterinary supervision and as a last resort [36]. These findings highlight the need for veterinarians to actively engage more directly with their clients and ensure that only correct amounts of drugs are dispensed and that farmers are aware of correct actions for their disposal.

Several farmers who were found to have Vibrostrep (dihydrostreptomycin) in their drug cupboards, when they no longer required the drug for specifically prescribed animal health treatments. One farmer had recently treated affected cows for *Actinobacillosis (*Woody Tongue), in most cases leftover of the drug occurs as the dose rate for the average weight of a dairy cow can vary and this is generally less than the required treatment dose on the manufacturers packaging. In this instance, the remaining drug volume was up to two doses and the discrepancy occurred due to requesting additional volume due to prolonging clinical signs and one having stopped treatment early due to disappearance of clinical signs. These answers demonstrate a clearer need for communicating the importance of correct drug disposal and antibiotic course completion. The VCNZ considers antibiotics as hazardous wastes and threat to public health, safety, and the environment, and requires their appropriate disposal [63], indicating that vets responsible for leaving excess drugs on farm could be in breach of their code of conduct. However, there seems to be a lack of regulatory oversight leaving both farmers and veterinarians confused as to appropriate antibiotic disposal. This lack of guidance is concerning when compared to the previously mentioned farmer responses regarding using left over antibiotics to treat other conditions which is a proven driver of resistance [2]. A key recommendation would be to follow the pharmaceutical society of New Zealand’s recommendation to “bring any unused antibiotics back to your pharmacy so they are disposed of safely” [53], however regulatory governance is crucial for the success of such initiatives.

### Antimicrobials and Food Safety

All farmers reported to know the application method of the veterinary medicines that they were using, they also all demonstrated good methods of record keeping including back up methods to avoid a breakdown in compliance. Furthermore, all could explain why the weight of the animal was important at a level suitable to show conceptual understanding with product withdrawal times and food safety. Moreover, farmers noted that if they did not know the application method, then they had strategies to identify the method of application. With only one farmer reporting that they did not know how they would find out the route of administration if they did not already know. These findings are different to those of Cresswell et al. [10] who found over 27% of farmers administered antibiotics via incorrect routes, although the reasoning behind these difference cannot be confirmed the importance of the RVM in providing an annual refresher in medicine administration cannot be overlooked. Interestingly, although none of the farmers interviewed in this study knew the full rationale behind application methods, all were able to make reference to why it had to be done a specific way, moreover all farmers expressed the importance of a dose rate again a difference found to those of [10].

Most farmers interviewed defined withdrawal times as the time “*it* [veterinary medicine] *takes to get out of the meat or milk*”, and even those farmers who did not consider contamination in their definition of withdrawal times spoke of keeping products out of the food chain, or the processing or manufacturing process. This is encouraging and shows the benefit of the shed inspections making farmers aware of their role within food safety. If veterinarians can capitalise on this knowledge, then it would aid in farmers feeling more involved in the overall process. These beliefs are shared by Scherpenzeel et al. [54], who documented that the outcomes of selective-dry-cow therapy was vastly improved when the dairy farmers felt positively about why they were undertaking this change of habit.

With regards to DCT, the survey was not specifically designed to explore its use, instead it was incorporated into questions regarding overall use of antimicrobials, such as appropriate treatment for mastitis or a cow at dry off. However, due to overwhelming evidence associated between AMU, AMR and DCT [19] it is worth noting that over half of the respondents, eight in total, had antimicrobial dry cow therapy on farm. This is interesting as this survey occurred post-calving season, and Aotearoa is a seasonal calving country. As such, DCT would not be required for at least six months in which time several of the inspected batches would be out of date. This fact compiled with several farmers openly admitted to keeping left over antibiotics from finished treatment to use in other animals, and for other disease conditions, is worthy of further investigation. One potential explanation of this is that farmers cannot receive financial reimbursement for unused antibiotics returned to veterinary practices, therefore all purchased drugs if not used must be disposed of [15]. This can be a difficult concept for farmers, perhaps government incentives would help to encourage farmers to do the right thing with regards to antibiotic disposal. This should be explored in future studies.

### RVM Procedure

Overall, farmers interviewed in this study found the RVM of use to their farm, however only one farmer was aware that it was a legal obligation, with the rest believing it was a practice-based decision. This is concerning as although McDougall et al. [37] suggest a trend of decreasing prevalence in some pathogenic organisms exhibiting AMR in Aotearoa, if farmers continue to be unaware of the greater implications of their behaviours around drug usage, and behavioural variation in AMU is allowed to continue as reported by Bryan and Hea [6], the downwards trend in level of AMR in Aotearoa could be reversed. The One Health approach to AMR requires stakeholders to contribute to cross-sectoral efforts to improve AMS, Degeling and Hall [11] discuss how a disconnect between how antibiotic use is conceptualized by dairy farmers and the way antimicrobial stewardship policies position agricultural AMR risks. If veterinarians make better use of the time allotted for the RVM consult, it could provide an opportunity to communicate the key themes of One Health and AMS to receptive clientele and encourage farmers to see themselves as part of the solution.

### Study design and limitations

The limitation of this study focuses primarily on geographic range as all dairy farmers who partook were from within the lower North Island of Aotearoa, as such meaningful conclusions cannot be extrapolated to refer to the entirety of the country. Furthermore, the sample size of this study was limited as this trial occurred at one veterinary practice and utilised all registered dairy clientele. The decision to undertake face to face interviews was chosen as, although postal or online survey provides ample opportunity for a high response rate, Mason [35] demonstrated, face-to-face interviews allow farmers to communicate their social experience and lived realities. To minimise disruption to the farmers the interview was conducted alongside routine dairy visitations and occurred shortly after their RVM consultation, despite this many responses were not as expected having participated in an RVM consultation shortly before. No farmers utilised the penultimate question regarding comments on the questionnaire negatively and those that did provide comments stated it was thorough and that it had made them think, although there is inherent bias to this question as the questionnaire was being conducted by their veterinarian. In expanding this study a similar format could be undertaken as it provided a chance for veterinarians to engage with their clientele in a different context. An anonymous postal survey is also worthy of consideration as Jones et al. [29] conducted anonymous postal surveys and received responses still in keeping with expected answers. However as Strang et al. [60] demonstrated dairy farmer response rate can be very low despite high veterinarian interest, as such an on-farm in person interview utilising the vet-client relationship was proven most effective as all fifteen farmers registered at the practice agreed to take part as long as it occurred during a routine call out. Further work regarding the questionnaire design is warranted, as although the farmers were appreciative of the speed of the questionnaire, aided by the number of binary questions. To trull engage with dairy farmer understanding of One Health and AMR and to conduct a fuller qualitative approach such as thematic analysis question redesign to more open ended questions is warranted, however as demonstrated by Hennessey and Barnett [24] this approach can be over and incorrectly utilised if the underlying theory is not fully engaged with. To overcome these study limitations the interview could be shortened and the questions, which are already split into sections, could be further classified with open questions at the start with leading questions included within a topic if required e.g. a study participant is not engaging with the theme of the question. The number of closed questions would therefore be decreased. For future recommendations the project should be rolled out across different vet practices and geographic locations across Aotearoa, thus increasing the number of farmers questioned and should explore the drivers for change and the cost of proposed interventions in dairy farmers.

In this study there were no inherit differences identified in the answers between farmer owner category e.g. share milker, manager etc., highest level of education, nor by ethnic identity. The small sample size is likely responsible for this. In this study there was relatively equal representation of farmer interviewed regarding gender identity which is unusual given the findings of Burton et al. [8] which showed men are still seen as the primary farmer.

## Conclusion

This pilot study has laid the groundwork for future research, involving larger surveys, into farmer knowledge, attitudes and practices around AMU and AMR as well as One Health within Aotearoa’s dairy industry. This study highlighted that there are notable gaps within dairy farmer understanding of AMU, AMR and One Health. Veterinarians need to do more to keep their clients informed of their important role and engagement within the One Health triad, especially regarding environmental aspects. There needs to be a push for veterinarians, farmers and industry representatives working together to embrace One Health, although the New Zealand antimicrobial resistance action plan provides an established and realistic pathway [42]. Simple solutions would be to encourage farmers returning unused drugs to their veterinarians for correct disposal, a task made easier if regulatorily required, and to actively engage farmers further within the AMU and AMR fields so that these end-product users do not feel disconnected from the process. Future work will focus on exploring farmer knowledge, attitudes and practices associated with antimicrobial stewardship will assist in operationalising the national AMR plan. To see this achieved, further work should focus on the intersection between quantitative and qualitative methodology with a mixed methods approach to ensure strides are made on this important global health threat.

### Supplementary Information

Supplementary Material 1.

## Data Availability

The anonymised data set collected in the project, is available upon reasonable request to the corresponding author.
