# Supplementary material for: Dairy farmer, engagement and understanding of One Health and antimicrobial resistance - a pilot survey from the lower north island of Aotearoa New Zealand
Source: One Health Outlook. 2024 Aug 1;6:14. doi: 10.1186/s42522-024-00107-7 (PMC11293148; doi:10.1186/s42522-024-00107-7)
Supplement: Supplementary file 1 — Supplementary Material 1. [file 42522_2024_107_MOESM1_ESM.docx]

Consent page

Are you the……

- Farm manager, Owner, Second in command.

Are you involved in the RVM consult?

- Yes, No

Do we have your permission to use this anonymised data?

- Yes, No

Do we have permission to access your Cornerstone records for anonymised data analysis?

- Yes, No

…………………………………………………………………………………………………………………………………………………………….

Age: 18 – 25

25 – 30

30 – 40

40 – 50

50 – 60

60+
 Prefer not to Say

Ethnicity: Māori

Pacific Islander

NZ European

NZ Asian

NZ Indian

Other (Please specify )

…………………………………………………………………………………………………………..

Prefer not to Say

Iwi:

Please state your iwi

…………………………………………………………………………………………………………..

Not relevant Prefer not to Say

What is your highest level of qualification: High school or equivalent

Undergraduate qualification

Post graduate qualification

Doctoral qualification

Other (Please specify )

…………………………………………………………………………………………………………..

Prefer not to Say

…………………………………………………………………………………………………………………………………………………………….

What do you think One Health means?

Do you know what is meant by Antimicrobial Resistance?

- What does it mean to you personally?

Are you aware of the New Zealand Veterinary Association (NZVA) antibiotic Traffic Light System?

- Yes, No

What do you think is the purpose of the antibiotic Traffic Light System?

Do you consider the NZVA traffic light system when discussing antibiotic treatment with your veterinarian?

- Yes, No

Do you consider antimicrobial resistance when requesting antibiotic treatment?

- Yes, No

Do you consider One Health when requesting antibiotic treatment?

- Yes, No

…………………………………………………………………………………………………………………………………………………………….

What is an RVM?

Why do we have an RVM?

What is the purpose of an RVM consult?

Do you understand the difference between the RVM and the RVM consult?

- Yes, No

Please explain the difference:

Do you find the annual RVM consult of value to your farm?

- Yes, No

Please explain your answer:

What is your opinion of the RVM consult?

What drugs are currently on your RVM list (From Memory)?

What drugs not on your RVM list do you use (From Memory)?

Do you have any drugs not on your RVM list, currently have on Farm?

How often do you perceive yourself using veterinary prescription drugs not on your RVM, on a yearly basis?

How often do you use drugs, not requiring veterinary prescription, nor from your RVM e.g. wormers etc?

What do you do with expired drugs?

When do you record your drug usage?

- Immediately (<5 minutes), within an hour, same day, same week, same month, I don’t record.

Where do you record your drug usage?

Who records the drug usage?

What percentage of the time do you record your drug usage?

- 100%, 90 – 100%, 80 – 90%, etc.

…………………………………………………………………………………………………………………………………………………………….

Are antibiotics your first line of treatment?

What conditions do you routinely treat with antibiotics?

How often do you request a culture and sensitivity?

- 100%, 90 – 100%, 80 – 90%, etc.
  - If so on what samples?

…………………………………………………………………………………………………………………………………………………………….

Do you know the required application method e.g. subcutaneous, for all drugs available on your RVM?

- If you do not, how would you find out this information?

What is meant by the term off label use?

- Different type of application (in muscle rather than under skin, other way around)
- Different dose/ higher/ lower dose than stated on RVM
- Different indication (AB used for sick calf rather than foot rot for example)
- Using two RVM drugs at the same time for an animal, i.e. antibiotic and NSAID
- All of the above
- Other (please specify)

What are withdrawal times and why are they necessary?

Have all staff members, expected to be able to administer drugs, been trained in medicine administration?

- Yes, No
  - If not why not? And if so, who conducted the training? Are written records kept of this?

Do you remember the withdrawal times of drugs available on your RVM?

- If you do not, how would you find out this information?

Why do different drugs have different administration routes?

Where would you find the correct dosages for products on your RVM?

Why are dose rates necessary?

…………………………………………………………………………………………………………………………………………………………….

Do you currently store Non-steroidal anti-inflammatories/pain relief e.g. Metacam/ketoprofen on farm?

How often is pain relief given alongside antibiotic treatment?

- How do you decided if it is needed or not? If not why not?

Are there any instances or conditions where you have administered pain relief on its own?

…………………………………………………………………………………………………………………………………………………………….

The following conditions are common diseases/disorders which affect dairy cows in New Zealand.
What drugs would you like to administer for each condition?

- Lame cow – foot rot
- Lame cow – White Line disease
- Lame cow – -sole ulcer
- Lame cow – other
- Coughing claves
- Scouring calves
- Navel infection
- Scouring adults
- Cows with high SCC
- Cows with mastitis
- Dirty cows – discharge post calving.

Would you ask veterinary advice for any of these conditions?

How do you “diagnose” conditions?

How do you decide what animals require treatments?

…………………………………………………………………………………………………………………………………………………………….

Do you think should shed inspections form part of the RVM process?

- Yes, No

Do you think vets should work closer with shed inspectors regarding drug storage and drug usage?

- Yes, No

Explain your answer

…………………………………………………………………………………………………………………………………………………………….

Do you have any anonymised feedback you would like to provide with regards to this questionnaire?

Do you have any anonymous feedback you would like to provide to the vet team regarding the RVM process?
